# Use of Asian selected agricultural byproducts to modulate rumen microbes and fermentation

**DOI:** 10.1186/s40104-016-0126-4

**Published:** 2016-12-15

**Authors:** Yasuo Kobayashi, Seongjin Oh, Htun Myint, Satoshi Koike

**Affiliations:** Lab of Animal Function and Nutrition, Research Faculty of Agriculture, Hokkaido University, Sapporo, 060-8589 Japan

**Keywords:** Agricultural byproduct, Fermentation, Fiber degradation, Methane mitigation, Microbiota, Plant secondary metabolites, Rumen

## Abstract

In the last five decades, attempts have been made to improve rumen fermentation and host animal nutrition through modulation of rumen microbiota. The goals have been decreasing methane production, partially inhibiting protein degradation to avoid excess release of ammonia, and activation of fiber digestion. The main approach has been the use of dietary supplements. Since growth-promoting antibiotics were banned in European countries in 2006, safer alternatives including plant-derived materials have been explored. Plant oils, their component fatty acids, plant secondary metabolites and other compounds have been studied, and many originate or are abundantly available in Asia as agricultural byproducts. In this review, the potency of selected byproducts in inhibition of methane production and protein degradation, and in stimulation of fiber degradation was described in relation to their modes of action. In particular, cashew and ginkgo byproducts containing alkylphenols to mitigate methane emission and bean husks as a source of functional fiber to boost the number of fiber-degrading bacteria were highlighted. Other byproducts influencing rumen microbiota and fermentation profile were also described. Future application of these feed and additive candidates is very dependent on a sufficient, cost-effective supply and optimal usage in feeding practice.

## Backgrounds

The rumen is a dense and diverse microbial ecosystem, capable of transforming fibrous plant material and non-protein nitrogen into valuable products, such as short chain fatty acids and microbial protein [1]. However, this fermentation process is accompanied by the synthesis of non-beneficial products such as methane and is not always efficient, due to the limited supply of essential nutrients and/or inadequate feed formulation. Therefore, particular attention should be paid to dietary regimens that optimize fermentation. Several dietary supplements have been proposed for such a purpose [2–6], targeting inhibition of methane and rapid ammonia release, and improvement of fiber degradation.

Inhibition of methane production and excess ammonia formation conserves dietary energy and proteins, respectively. These effects were observed after supplementation with antibiotics [4] and halogenic chemicals [7], the majority of which have now fallen out of favor due to global concerns regarding food safety and environmental burden. Therefore, alternative agents are required, preferably naturally occurring materials such as plant resources [3, 8]. The main components, most of which are plant secondary materials, have been screened out. They have ecological functions as chemical messengers between plants and the environment, often exhibiting antimicrobial activity [9]. Such alternatives have been actively explored, especially since growth-promoting antibiotics were banned in Europe in 2006.

Fiber digestion is preceded by fiber-digesting rumen microbes, mainly bacteria [10]. Therefore, preferential activation of fibrolytic rumen bacteria is important. Bacterial growth can be stimulated by vitamins, amino acids, branched chain fatty acids and other nutrients. Additionally, the use of easily degradable fiber as a strategy has been known since the 1980s [11–13]. Evaluation of supplements as boosters for fiber degradation should include the determination of fiber digestibility as well as the analysis of rumen bacterial abundance and activity. A mechanistic understanding of expected events would confirm theoretical knowledge, making supplement use more acceptable to farmers. Materials that have been proposed in the last decade include agricultural by-products deemed safe, cost-effective and easily acceptable among farmers and product consumers.

This review describes selected agricultural byproducts that are available in the Asian region as potent feed or additive candidates for the above purposes. Characteristics, actions and benefits of such agricultural byproducts are discussed from the viewpoint of modulation of rumen microbiota and fermentation.

### Selected byproducts containing plant secondary compounds as inhibitors of formation of non-beneficial fermentation products

#### Cashew byproduct

Cashew nut shell liquid (CNSL), a byproduct of cashew nut production that accounts for about 32% of the shell, has many industrial applications and is used as a raw material for products such as paints, brake linings, lacquers and coatings [14]. The global production of CNSL is estimated at 450,000 metric tonnes per year [15], providing a readily available supply of CNSL. Vietnam and India are major CNSL-producing countries. This liquid also exhibits a wide range of biological activities, as it contains compounds with antimicrobial [16], antioxidative [17] and antitumor [18] properties, represented by anacardic acid, cardanol and cardol, which are all salicylic acid derivatives with a carbon-15 alkyl group. These phenolic compounds, especially anacardic acid, are reported to inhibit a variety of bacteria [19]. Proportions of these alkyl phenols in CNSL vary with producing area (cultivar) and deshelling process (heating). Therefore, the function of CNSL as a rumen modifier can also vary with these factors, as indicated in Tables 1 and 2.

**Table 1:** Effect of selected agricultural byproducts containing anacardic acid and other phenolics on dry matter (DM) digestibility and rumen fermentation parameters

Byproduct, origin	Description	Phenolics, % in weight			Reference	Test by	Dosed at	DM digestibility, %	Total VFA, mmol/dL	Inhibition, %		Reference
		Anacardic acid	Caldanol	Caldol						Methane^a^	Ammonia	
Cashew shell, India	Heated^b^	-	71.4	14.4	[21]	Batch culture	0.5 mg/mL	-	ns	9.2	-	[21]
	Raw	57.7	8.2	19.9	ibid.		ibid	-	ns	56.9	-	ibid.
	Raw	ibid.	ibid.	ibid.	ibid.	RUSITEC	0.2 mg/mL	↑	ns	70.1	16.5	ibid.
	Raw	ibid.	ibid.	ibid.	ibid.	Feeding (dry cow)	0.32% of DMI	ns	ns	19.3–38.3	ns	[22]
	Raw	ibid.	ibid.	ibid.	ibid.	Feeding (milking cow)	ibid.	ns	ns	12.7	ns	Shinkai et al. unpublished
	Raw	ibid.	ibid.	ibid.	ibid.	Feeding (sheep)	ibid.	ns	ns	61.4	43.0	Suzuki et al. unpublished
Cashew shell, Tanzania	Raw	-	-	-	[25]	Batch culture	0.17 mg/mL	-	ns	17.8	-	[25]
Cashew shell, Brazil	Heated	-	62.9	13.4	[26]	Feeding (milking cow)	0.11% of DMI	ns	-	8.0	-	[26]
	Raw	64.9	1.2	13.3	[29]							
Cashew shell, Brazil	Heated	-	73.3	19.4	[28]	Feeding (milking cow)	0.036% of DMI	ns	-	-	-	[28]
	Raw	49.3	30.5	20.2	[27]							
Ginkgo fruit, Japan	Cultivar A	85.0	2.3	12.7	Oh et al.	Batch culture	3.2 mg/mL	-	ns	85.7	42.0	Oh et al. unpublished
	Cultivar B	86.8	2.3	10.9	unpublished	ibid.	4.5 mg/mL	-	ns	65.9	46.0	ibid.
						RUSITEC	3.2 mg/mL	ns	ns	47.3	53.7	ibid.
Ginkgo leaf, Korea	Unspecified	-	-	-		Batch	1.0 mg/mL^c^	ns	ns	46.7	-	[30]

-, No data available

ns, Not significantly changed

↑, Significantly increased

^a^Calculated on basis of CH4 ml for in vitro test and of CH4 g/kg DMI for in vivo test, respectively

^b^Heat was used in deshelling process

^c^g of extract/mL (not calculable as original leaf)

**Table 2:** Effect of selected agricultural byproducts containing anacardic acid and other phenolics on rumen microbial abundance determined by quantitative PCR

Byproduct	Main compound involved	Tested by	Dosed at	Abundance of rumen microbe, relative % to total bacteria															Reference
				Pro	Meth	Fu	Fs	Rf	Ra	Me	Sr	Sd	Tb	Sb	Pr	Pb	Rm	Al	
Cashew shell	Anacardic acid	RUSITEC	0.2 mg/mL	↓^a^	ns	-	↓	↓	ns	↑	↑	↑	↓	ns	↓	↓	ns	↑	[21]
		Feeding (dry cow)	0.32% of DMI	ns^a^	ns	-	ns	↓	↓	-	↑	↑	↓	-	↑	-	-	↑	[22]
		Feeding (milking cow)	0.33% of DMI	-	ns	ns	↓	ns	ns	ns	ns	ns	ns	ns	ns	ns	ns	ns	Shinkai et al. unpublished
		Feeding (sheep)	0.32% of DMI	↓^a^	-	-	ns	ns	ns	ns	ns	ns	ns	ns	ns	ns	ns	ns	Suzuki et al. unpublished
Ginkgo fruit	Anacardic acid	Batch culture, culivar A	3.2 mg/mL	-	↑	-	ns	ns	ns	↑	↑	↑	↓	↑	ns	↑	ns	ns	Oh et al. unpublished
		Batch culture, cultivar B	4.5 mg/mL	-	↑	-	ns	ns	ns	ns	ns	↓	ns	↑	ns	↑	ns	↓	ibid.
		RUSITEC, cultivar A	3.2 mg/mL	↓	↓	↓	↓	↓	↓	↑	↑	↑	↓	ns	↑	↓	↑	↑	ibid.
Ginkgo leaf	Unspecified	Batch culture	1.0 mg/mL^b^	↓	↑	-	↑	↓	↓	-	-	-	-	-	-	-	-	-	[30]

Pro protozoa, Meth methanogen, Fu fungi, Fs *Fibrobacter succinogenes*, Rf *Ruminococcus flavefaciens*, Ra *Ruminococcus albus*, Me *Megasphaera elsdenii*, Sr *Selenomonas ruminantium*, Sd *Succinovibrio dextrinosolvens*, Tb *Treponema bryantii*, Sb *Streptococcus bovis*, Pr *Prevotella ruminicola*, Pb *Prevotella bryantii*, Rm *Ruminobacter amylophilus*, Al *Anaerovibrio lipolytica*

-, No data available

ns, Not significantly changed

↑, Significantly increased

↓, Significantly decreased

^a^Value were obtained by direct counting

^b^g of extract/mL (not calculable as original leaf)

An early study by Van Nevel et al. [20] first indicated that anacardic acid could be used as a propionate enhancer in the rumen. Anacardic acid is found in cashew and ginkgo trees, particularly in their seeds. As cashew is the more abundant plant material, it is considered a more useful source of anacardic acid. The main action of anacardic acid and related phenolics is a surfactant action that inhibits mainly Gram-positive bacteria [16] lacking an outer membrane. Such cells are physically disrupted by anacardic acid. This selective inhibition of Gram-positive rumen bacteria might result in the alteration of rumen microbiota and fermentation products.

Indeed, Watanabe et al. [21] first indicated that unheated CNSL dramatically reduced methane production while increasing propionate production in batch cultures. They also reported that CNSL reduced methane levels in a rumen simulation technique (RUSITEC) fermenter, accompanied by drastic alterations in rumen microbiota. Quantitative polymerase chain reaction (PCR) demonstrated that formate and/or hydrogen producing bacteria decreased in abundance, while succinate and/or propionate producing bacteria increased with CNSL supplementation. In feeding experiments using cattle, we observed a similar response to CNSL [22]; specifically, a reduction in methane emission (19-38%) accompanied by alteration in the ruminal abundance of bacterial species responsible for methane and propionate production, causing a shift in hydrogen flow [23]. However, as expected, alterations of microbiota and fermentation profile in these feeding studies were less pronounced than those in *in vitro* studies. In feeding experiments using sheep, microbial and metabolic alterations were also observed, although alterations in the abundance of bacterial and archaeal members in sheep rumen (Suzuki et al. unpublished results) were not the same as those observed in cattle rumen (Su et al. unpublished results). In fact, in response to CNSL feeding, groups belonging to Proteobacteria, relatives of *Succinivibrio* and *Succinimonas*, showed increased levels in the rumen of cattle and sheep, while increases in *Methanomicrobium mobile* and *Methanobrevibacter wolinii* were respectively observed in the rumen of cattle and sheep.

As CNSL administration did not adversely affect digestibility in either cattle or sheep, this agricultural byproduct can be recommended for use as a potent methane-inhibiting and propionate-enhancing agent, due to its effects on rumen microbiota. However, the long-term effects of CNSL should be evaluated for practical application, as was emphasized for the ionophore monensin [24], which showed a reduction in efficacy with increased feeding period duration.

Later *in vitro* and *in vivo* studies on CNSL do not wholly support the above favorable results, due to the low level of CNSL supplementation and heat treatment for CNSL preparation (Table 1). Although CNSL supplementation decreased methane production, inhibition was only 18% [25], while it was 57% in the similar batch culture system used in our study [21]. CNSL feeding to dairy cows decreased methane emission by only 8% [26]. The differences between these later results and our initial ones might be the quantity and quality of CNSL. Danielson et al. [25] tested 3 times lower supplementation level of CNSL than the level examined by Watanabe et al. [21], and Branco et al. [26] used heat-processed CNSL that contains cardanol as a main phenolic compound instead of the most potent phenolic, anacardic acid [27–29]. Microbial response was clearly different between these studies. Our MiSeq data in our RUSITEC study demonstrated drastic alteration of microbial community structures: for eubacteria, a higher detection frequency of Veillonellaceae and Succinivibrionaceae and lower frequency of the Ruminococcaceae, and for archaea, a higher frequency of Methanomicrobiaceae and lower frequency of Methanobacteriaceae (Kobayashi et al. unpublished results). Therefore, this cashew byproduct should be used in unheated form at an optimized supplementation level. Of alkylphenols present in CNSL, anacardic acid is most functional but decarboxylated and converted to caldanol by heating and long exposure to oxygen. Therefore, preparation and storage of CNSL are important to maintain its functionality.

Recently, we found that CNSL feeding improved antioxidative status in cattle, causing higher free radical scavenging activity and lower lipid peroxidation products in the rumen and blood serum (Konda et al*.* unpublished results). Although the mechanisms involved in these changes are not yet clear, anacardic acid possessing antioxidative activity [17], can affect theses parameters directly and/or indirectly through alteration of rumen microbiota and their fermentation products.

#### Ginkgo byproduct

Another source of anacardic acid is the ginkgo plant, grown widely among Far-East countries such as China, Korea and Japan. Industrial uses of ginkgo are its leaves for medicinal use (China) and its nuts for food (Japan). Leaf extracts for medicinal use are even exported to European countries and also evaluated as a rumen modifier [30]. Ginkgo fruit is a byproduct in the process of ginkgo nut separation (unsuitable for human food use due to its peculiar smell), yielding ca. 2,600 metric t/yr in Japan, accounted for 230% of nut production [31]. Therefore, biomass of ginkgo fruit is much smaller in comparison with CNSL. In this regard, use for feed additive might be limited locally.

The main phenolic of ginkgo is anacardic acid, but it has different alkyl groups in comparison with those of cashew (C13:0, C15:1 and C17:1 for ginkgo vs. C15:1, C15:2 and C15:3 for cashew). An *in vitro* evaluation of ginkgo fruit extract as a rumen modifier using batch and RUSITEC systems showed that the extract decreased methane production in a dose-dependent manner and microbial responses were similar to those observed for CNSL (Tables 1 and 2), though such potency depends on the cultivar (Oh et al*.* unpublished results). The most potent phenolic for bacterial selection was anacardic acid, in particular monoenoic (15:1) anacardic acid. Our MiSeq data suggest that ginkgo fruit extract greatly modulates the microbiota of RUSITEC (Oh et al*.* unpublished results) similarly to what was found for CNSL supplementation.

Both CNSL [21] and ginkgo fruit extract (Oh et al*.* unpublished results) decrease ammonia concentration in RUSITEC. Since both inhibit the growth of proteolytic, peptidolytic and deaminating rumen bacteria in pure culture, feeding of these extracts may spare dietary protein, peptide and amino acid. In fact, the growth of hyper ammonia-producing rumen bacteria was markedly inhibited by either the form of anacardic acid contained in CNSL or ginkgo fruit extract (Oh et al*.* unpublished results). Manipulation of protein and amino acid degradation is important, because excreted ammonia could be the source of nitrous oxide, which has much higher potential for global warming than methane. Also, decreased ammonia level in the rumen, but not lower than 5 mgN/dL to ensure microbial protein synthesis [32], may improve feed nitrogen economy. Since ginkgo fruit has not been tested in a feeding study, *in vivo* evaluation is to be made on rumen and animal responses including palatability of the diet to which ginkgo fruit is supplemented.

#### Tea byproduct

China is one of the biggest tea producers globally. Tea seed meal after oil extraction has previously been considered worthless. However, saponins contained in the tea seed meal have been found to exert beneficial antiprotozoal and antimethanogenic effects through surfactant action [33]. Significance of tea saponins and other source plants such as yucca and quillaja for the use of ruminant feed has been demonstrated [33, 34]. Table 3 shows functionality of saponins of tea seed, tea seed meal and other source plants (Thai blueberry, fenugreek, and mangosteen). A series of studies on tea seed saponins revealed that the addition of tea seed saponins to *in vitro* cultures killed up to 79 % of protozoa. Moreover, *in vivo* experiments (feeding of tea seed saponin to lambs at 3 g/d) showed that the relative number of rumen protozoa to rumen bacteria was reduced by 41% after 72 d of tea saponin administration [35]. Using denaturing gradient gel electrophoresis (DGGE) analysis, a significantly lower diversity in protozoa was reported [36], indicating that the antiprotozoal activity of tea saponins might not be transient. Although an exception was observed by Ramirez-Restrepo [37], negative effect of tea saponins on rumen protozoa is consistent regardless of *in vitro* and *in vivo* conditions, and considered as one of main factors to modulate rumen fermentation in relation to bacterial and archaeal changes as discussed below.

**Table 3:** Effect of selected agricultural byproducts containing saponins and other phenolics on dry matter (DM) digestibility, rumen fermentation parameters and microbial abundance

Byproduct	Main compounds involved	Tested by	Dosed at	DM digestibility, %	Total VFA, mmol/dL	Inhibition, %			Abundance, relative %					Reference
						Methane	Ammonia	Protozoa	Meth	Fungi	Fs	Rf	Ra	
Tea seed/seed meal	Saponins	Batch culture	0.4 mg/mL	-	ns	8.0	-	51.3	ns	↓	↑	ns	-	[38]
		Feeding (sheep)	3 g/d^a^	-	ns	10.6	13.2	43.2	ns	ns	↓	ns	ns	[36]
		Feeding (steer)	0.24–0.38% of DMI	-	ns	15.6	-	ns	ns	-	↑	↓	↑	[37]
		Feeding (growing lamb)	0.41% of DMI	-	↑	27.5	ns	41.1	ns	ns	-	ns	ns	[35]
Thai bllueberry seed	Saponins	Feeding (goat)	0.8–24% of DMI	ns	ns	2.2–8.0	ns	ns	-	-	-	-	-	[46]
Fenugreek seed	Saponins	Batch culture	0.14–0.29 mg/mL	-	ns	1.8–2.0	-	15.0–39.0	↓	↓	↑	↑	-	[41]
Mangosteen peel	Saponins, tannins	Feeding (dairy cow)	100–300 g/d^a^	-	-	5.5–13.8	-	20.5–47.1	↓	ns	ns	ns	ns	[47]
Eucarypus leaf meal	Cineol, cryptone etc.	Feeding (swamp buffalo)	0.7–2.0% of DMI	ns	↑	8.4–13.9	12.7–33.9	5.5–22.0	-	-	-	-	-	{51}

Meth methanogen, *Fs Fibrobacter succinogenes*, Rf *Ruminococcus flavefaciens*, Ra *Ruminococcus albus*

-, No data available

ns, Not significantly changed

↑, Significantly increased

↓, Significantly decreased

^a^Dosage could not be expressed as % of dry matter intake (DMI) due to lack of data on feed intake

The effect of tea saponins on the ruminal abundance of methanogenic archaea was not significant, while they drastically decreased the expression of the methyl coenzyme M reductase gene (*mcrA*) in the rumen [38]. This suggests that selective inhibition of methanogens might be involved in the antiprotozoal action. Using defaunated and refaunated sheep, Zhou et al*.* [36] showed that tea saponins reduce methane production by inhibiting protozoa, most likely in coordination with their suppressive effects on protozoa-associated methanogens. Indeed, the presence and functional significance of protozoa-associated methanogens has been demonstrated [39, 40].

Saponins alter rumen microbial community with a decrease in protozoa and fungi and increase in *Fibrobacter succinogenes* [38, 41]. The latter can compensate for fiber digestion possibly depressed by the decreased number of fungi, leading to a fermentation change toward less methane and more propionate, since protozoa and fungi produce hydrogen, while *F. succinogenes* produces succinate as a propionate precursor. Recently, Belanche et al*.* [42] reported decreased diversity in the archaeal community by supplementation with ivy fruit saponins in RUSITEC fermenter: *Methanomassilicocaaceae* is substituted by *Methanobrevibacter*, a theoretically less active community member even though it is predominant in the rumen [43]. From these reports, it is apparent that the mechanism involved in the modulation of rumen fermentation by saponins remains to be fully characterized. Ruminal responses could differ depending on saponins that occur in a number of plants and comprise a variety of molecules. Tea saponins are, as indicated by a review article [34], one of the promising rumen modifier without negative influence on feed intake and digestibility if supplemented properly (3–5 g/d for goats and lambs).

Tea byproducts also contain catechin that can increase the proportion of unsaturated fatty acids in goat meat [44], presumably through alterations in the rumen microbiota. Another beneficial action of tea catechin is to improve antioxidant status of beef, once the catechins are ingested and absorbed by the animal. This was speculated by direct addition of tea catechins to beef [45].

#### Other byproducts

Other materials potentially modulating rumen fermentation are also shown in Table 3. Fenugreek is cultivated in western and southern Asian regions, where it is used as a spice, seasoning, fragrance in the form of sprouts, and is also known as a source of saponins. Fenugreek seed extract rich in saponin (0.29 mg/mL of diluted rumen fluid) inhibits growth of protozoa and fungi and increases growth of fibrolytic bacteria, leading to 2% decrease of methane production *in vitro* [41], awaiting a feeding assessment.

The seeds of Thai blueberry, *Antidesma thwaitesianum* Muell. Arg., containing condensed tannin, were evaluated as a ruminant feed [46]; goats fed the diet with this meal from the wine and juice industry (inclusion of 0.8–2.4% in DM) did not show any differences in feed intake, digestibility, ruminal pH or ammonia-nitrogen, while they showed a dose-dependent shift in short chain fatty acid production toward more propionate and less acetate and butyrate. Methane production linearly decreased (up to 8%) and nitrogen retention linearly increased (up to 45%) with seed meal supplementation level. Therefore, this byproduct might be an effective modulator of rumen fermentation and ruminant nutrition, though the mechanisms involved are not clear.

Feeding of mangosteen peel powder to lactating cows (300 g/d) can decrease methane production by 14% with a drastic decrease of rumen protozoa, while other representative rumen microbes are not affected [47]. Since mangosteen contains not only saponins but also condensed tannins, microbial and fermentation changes might be due to these two secondary metabolites.

Polyphenols in chickpea husk (abundantly available in southern and western Asia) exert antibacterial activity against mainly Gram-positive bacteria [48]. Rats fed chickpea husk at 5% level showed an altered hindgut bacterial community based on different DGGE banding patterns [49]. The authors also found that chickpea husk extract exhibited anti-oxidative activity measured as free radical scavenging activity and lipid peroxidation. In fact, rats fed chickpea husk had lower thiobarbituric acid reactive substance (TBARS) values in their blood plasma, suggesting the potency of this byproduct as a health-promoting agent in animals [49]. These favorable effects of chickpea husk are considered to be due to the presence of tannins that could have different impact depending on molecular species (*i.e.* source plants, cultivars and growing region) [50].

Asia is the origin of many plants that are sources of essential oils. As a byproduct of essential oil, leaf meal of *Eucalyptus camaldulensis* is paid attention due to the ability to decrease rumen ammonia level (by 34%) when fed to swamp buffaloes (120 g/d) possibly through the action of 1,8-cineol [51]. Therefore, it is proposed as another possible manipulator of protein and amino acid degradation in the rumen, which might save feed nitrogen. Since essential oils are generally expensive, their byproducts (residue of oil extraction) such as the above leaf meal is one option recommended for practical use.

New additive candidates from Asian agricultural byproducts have been explored for the use to decrease rumen methane and ammonia, in which *in vitro* evaluation is often used for initial screening. This evaluation is quick, quantitative, and very useful to define mechanisms involved in the efficacy of candidate material. However, as *in vitro* effect is always higher than *in vivo* effect, final recommendation is to be made after detailed evaluation by a series of feeding studies.

### Easily digestible fibers as boosters of fiber degraders

#### Chickpea and lablab bean husks

Fibers are not always efficiently degraded in the rumen due to complexity of fiber structure and components and less well optimized rumen microbiota. Recently, some easily degradable fibers have been proposed to modulate rumen microbiota toward quick optimization of developing fiber-degrading consortia [52]. We have found that husks from a few species of local beans (chickpea and lablab bean) show high potency in improving rumen fermentation [52, 53]. The functionality of these husks is summarized in Table 4. These fiber sources are considered a replaceable fibrous feed, as well as a booster of the degradation of the main forage. Indeed, these fiber sources can be characterized as easily digestible [11, 12].

**Table 4:** Stimulation of growth of representative fibrolytic rumen bacteria by bean husks

		Rumen bacterial colonization^a^						Rumen bacterial abundance^b^		
Fiber or husk, origin	H/C ratio^c^	By qPCR, × 10^7^/mL			By clone library, %			By qPCR, × 10^7^/mL		
		Fs	Rf	Ra	Fs	Rf	Ra	Fs	Rf	Ra
Beet pulp, Japan	1.53	0.2	5.0	0.1	-	-	-	-	-	-
Rice straw, Japan	0.68	747.4	36.7	19.0	3.2	0.0	0.0	30.9	1.2	0.8
Chickpea, Myanmar	0.06	476.3	72.4	7.2	6.5	0.0	3.2	229.1	1.9	0.3
Lablab bean, Myanmar	0.38	1044.0	27.5	91.6	1.4	0.0	2.8	371.5	3.2	6.0

Data are based on Fuma et al. [52] and Ngwe et al. [53]

^a^Each fiber source was incubated for 24 h in the rumen and colonized bacteria were quantified by quantitative PCR (qPCR)

^b^Bacteria quantified were *Fibrobacter succinogenes* (Fs), *Ruminococcus flavefaciens* (Rf) and *Ruminococcus albus* (Ra)

^c^Hemicellulose/cellulose ratio indicates the degree of complexity of fiber structure

Easily digestible fiber sources might promote the rapid growth of fibrolytic microbial biomass, which in turn facilitates the digestion of the other fiber in the rumen. Ammonia-treated barley straw and hay [11] have been used as sources of easily digestible cellulose and/or hemicellulose. Unmolassed sugar beet pulp [12, 54], citrus pulp and dried grass [12], ammonia-treated rice straw [55] and soybean hull [56] are also sources of easily digestible fiber. However, their properties have not been fully characterized, especially in relation to the activation of fibrolytic rumen microbes.

It is imperative to determine whether the rumen bacteria that are activated by supplemental fiber correspond to the bacteria that are responsible for main forage digestion [53]; otherwise, this fiber cannot be considered a booster of main forage degradation. In this regard, local bean husks seem ideal for the enhancement of rice straw digestion, as they increased the ruminal abundance of the representative fibrolytic bacterium *Fibrobacter succinogenes* [53], whose importance in the degradation of grass forage such as rice straw is extensively studied [57–64] and widely accepted [65, 66]. Sugar beet pulp, another easily digestible fiber that finds popular use in several countries, was eliminated by initial screening due to its failure to activate *F. succinogenes* [53].

Specific activation of *F. succinogenes* by selected materials (chickpea husk and lablab bean husk) was confirmed in a series of *in situ* and *in vitro* studies [52, 53]. Quantitative PCR indicated that these fiber sources were heavily colonized by *F. succinogenes*. Pure cultures of several different strains of *F. succinogenes* revealed growth stimulation after addition of the bean husks as the sole carbon substrate.

Finally, a digestion trial, in which each type of husk was supplemented at 10%, was employed to evaluate them as digestion boosters for a rice straw-based diet [53]. The digestibility of acid detergent fiber was 3.1–5.5% greater in diets supplemented with chickpea husk or lablab bean husk than in the control. Total short chain fatty acid levels were higher in sheep fed lablab bean husk-supplemented diet than in sheep fed other diets, while acetate levels were higher in lablab bean husk-supplemented diet than in the control diet. Ruminal abundance of *F. succinogenes* was 1.3–1.5 times greater in diets supplemented with chickpea husk or lablab bean husk than the control diet. These results suggest that bean husk supplementation might improve the nutritive value of a rice straw diet by stimulating the growth of fibrolytic bacteria, represented by *F. succinogenes*. Regarding the use of chickpea husk, selection of cultivar may be important, because some show a higher content of tannin (*e.g.* chickpea husk from western Asia) that can inhibit fibrolytic bacteria and their enzymes.

#### Soybean hull

Soybean hull (soybean husk) is one of a number of popular feed ingredients that are partly interchangeable with main forages (up to 25–30% of dry matter intake) for lactating dairy cows without negatively affecting fermentation, digestion or production performance [67]. Soybean hull activated representative rumen cellulolytic and hemicellulolytic bacteria in a pure culture study, and growth stimulation of *Prevotella ruminocola* was notable after incubation with the water soluble fraction of soybean hull (Yasuda et al*.* unpublished results). Therefore, this familiar feed should be reevaluated for its potency in activating specific but important rumen bacteria and further examined to optimize its usage. Soybean hull also has unidentified functions that can modulate hindgut microbiota and fermentation in monogastric animals. Rats fed a diet containing 5% soybean hull showed higher abundance of lactobacilli, leading to a higher lactate level and lower pH in the cecum in comparison with a control diet containing 5% cellulose, and this was partly explained by the presence of oligosaccharides in soybean hull (Htun et al*.* unpublished results). These results indicate availability of this material for non-ruminant animals, even companion animals such as dogs, as reported by Cole et al*.* [68], who valued the hull as a dietary fiber source.

## Conclusions

Representative materials and components showing rumen modulatory effects, many of which can be obtained from Asian agricultural products, were introduced in this review. We focused on inhibition of methane production and protein degradation, and on stimulation of fiber digestion. Evaluation of such byproducts and their components should include mechanistic analyses together with practical feeding trials. Since the availability of candidate byproducts may depend on the region, cost-effective use of individual byproducts should be developed locally. Once the functional potency and a sufficient supply of candidate byproducts can be globally confirmed, these byproducts hold promise as rumen modulators to improve rumen fermentation and enable safer, healthier, more efficient and environmentally friendly production of ruminant animals.

## Abbreviations

CNSL: Cashew nut shell liquid
DGGE: Denaturing gradient gel electrophoresis
PCR: Polymerase chain reaction
RUSITEC: Rumen simulation technique
TBARS: Thiobarbituric acid reactive substance
